# Variational Optimization for Sustainable Massive MIMO Base Station Switching

**DOI:** 10.3390/s24020520

**Published:** 2024-01-14

**Authors:** Aida Al-Samawi, Liyth Nissirat

**Affiliations:** Department of Computer Networks, College of Computer Sciences and Information Technology, King Faisal University, Al-Ahsa 31982, Saudi Arabia; lnissirat@kfu.edu.sa

**Keywords:** sustainable networking, green communications, massive MMO

## Abstract

Massive MIMO networks are a promising technology for achieving ultra-high capacity and meeting future wireless service demand. Massive MIMO networks, on the other hand, consume intensive energy. As a result, energy-efficient operation of massive MMO networks became a requirement rather than a luxury. Many NP-hard concavity search algorithms for optimal base station switching on-off scheme have been developed. This paper demonstrates the formulation of massive MIMO networks energy efficiency as a constrained variational problem. Our proposed method solution’s uniqueness and boundedness are demonstrated and proven. The developed system is a total energy optimization problem formulation. Furthermore, the order in which the base stations are switched on and off is specified for minimal handover overhead signaling and fair user capacity sharing. Results showed that variational optimization yielded optimal base station switching on and off with considerable energy saving achieved and maintaining the user capacity demand. Moreover, the proposed base station selection criteria provided suboptimal handover overhead signaling.

## 1. Introduction

Massive MIMO networks, heralded as promising heterogeneous technologies, are key to meeting future wireless system capacity demands. These networks typically consist of a MIMO macro base station (mBS) with multiple antennas, complemented by numerous smaller base stations, often micro base stations. However, their high energy consumption poses a significant challenge for their widespread adoption in future networks. This concern is heightened by the rising costs of energy, increased greenhouse gas (GHG) emissions, and the depletion of energy resources.

To illustrate the importance of energy efficiency in massive MIMO deployment, Auer et al. [1] reported on the total power consumption of various base station types under full load. For example, a macro base station with two antennas consumes approximately 1350 W at full load, with 55% to 60% of this power used by the power amplifier. In contrast, micro base stations with three sectors consume about 145 W at full load, with 30% of this power used by the power amplifier. Modern MIMO systems often employ remote radio heads to enhance base station efficiency and coverage, but this addition increases the total power consumption in the network. A remote radio head with two antennas, for instance, can consume up to 755 W in total power [1].

The power consumption in a network varies with the network density, population density in the area, and the type of traffic. In densely populated urban areas with over 1000 citizens per square kilometer, a network comprising 19 sites with three sectors each and a 2 × 2 MIMO transmission can consume over 4 kWh/m^2^ with no load, potentially rising to over 5 kWh/m^2^ at full load. In contrast, rural and suburban areas with less than 400 citizens per square kilometer may see consumption rates of 0.35 kWh/m^2^ at low load, increasing to 0.45 kWh/m^2^ at full load.

Moreover, Fehske et al. [2] projected that the total GHG emissions from mobile networks could reach 235 MtCO2-eq in 2020, a more than 270% increase from 2007 levels. This accounts for 4% of global carbon emissions in 2020, with projections suggesting a potential rise to 7% in a worst-case scenario by 2025 [3]. These alarming trends in GHG emissions and power consumption have spurred researchers to develop algorithms for energy-efficient deployment of massive MIMO systems. Base stations, as the major power consumers in wireless networks, are estimated to account for around 70% of the network power [4]. Variations in the spatial distribution of users within a base station’s coverage area often result in some stations operating under partial load. Consequently, switching off base stations during periods of low traffic can significantly enhance the network’s overall energy efficiency. However, in dense networks, balancing energy reduction through base station on-off switching while maintaining service quality is challenging.

To address this complexity, various approaches have been developed for optimizing base station switching in different network topologies. These include convex optimization using game theory combined with integer programming techniques for massive MIMO base station on-off switching [5], reinforcement learning algorithms for optimal base station settings based on Radio Environment Maps (REM) [6], and access point switching strategies based on the number and location of mobile users in cell-free massive MIMO networks [7]. Additionally, Jose A. Ayala-Romero et al. [8] approached HetNet base station switching as a finite horizon Markov Decision Process, optimizing it using certainty equivalent control (CEC) dynamic programming techniques. Distance constraint hard core point processes have been proposed for energy-balanced base station switch-offs [9], and dynamic programming techniques have been developed for selecting radio frequency chains and optimal active base stations [10].

Other algorithms aimed at enhancing energy efficiency in MIMO systems include the use of statistical channel state indicators [11], load adaptive energy efficiency strategies [12], network planning and optimal deployment [13,14,15], and SINR constraints and power adaptation techniques [16,17]. The authors in [18] focused on dynamic optimization for minimizing average total power costs in time-varying systems under imperfect channel conditions, employing Lyapunov optimization and fractional programming for robust sparse beamforming. This approach demonstrates rapid convergence and balances network power consumption against queue latency. It also addresses challenges of imperfect channel state information (CSI) and provides insights for sustainable network operation in real-world conditions. Meanwhile, research highlighted in [19] emphasizes the importance of managing base station energy consumption, including cell switch-off techniques, and explores machine learning’s role in optimizing energy usage. This research delves into various approaches to reduce power consumption, such as utilizing green energy sources and modifying base station coverage based on load levels. In this paper, we propose a novel variational base station switching on-off algorithm for energy-efficient massive MIMO network operation. First, we show that multi-base station massive MIMO network energy efficiency is a constraint variational problem. Then, we develop a capacity-aware power model of the massive MIMO cluster. In the developed power model, we will introduce a function that accounts for the optimal number of active base stations that would provide the required total bandwidth demand to the users. The Euler–Langrange optimization criteria are then applied in this work to derive deferential equation that estimates the optimal number of active base stations at any given time based on the Lagrangian multipliers.

The next section discusses the massive MIMO and base station topology assumed in this work and the derivation of bandwidth-aware total network power consumption. The formulation and optimization of active base station constraint variational problem are also presented in the Section 2, followed by results and discussion in the Section 3. Conclusive remarks are presented in the Section 4.

## 2. Multi Base Station Massive MIMO Power Model

**Figure 1:**
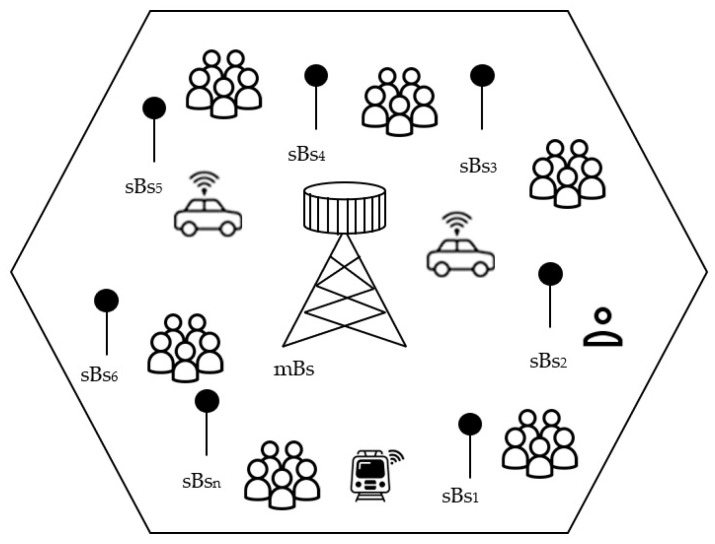
Proposed massive MIMO system.

Massive MIMO systems consist of a multi-antenna macro base station, referred to as mBs in this paper, with multiple micro base stations, referred to as sBs, as depicted in Figure 1. We assume that mBs is active permanently and sBs are switchable on-off. Furthermore, sBs have sleep mode and deep sleep mode, differentiated through their power consumption and awaking methods. Furthermore, we assume that sBs adopt Orthogonal Frequency Multiple Access OFDMA. In OFDMA, each sBs spectrum is divided into channels and each channel is further divided into subcarriers. The association of users’ with sBs is based on the bandwidth acquired by the user, the distance vector for each user from the desired serving sBs, and the availability of bandwidth in the sBs. User *i* association to sBs *j* is formulated as association matrix ($A_{m}$), where each element of the matrix is defined as follows:

(1) $$A_{m}^{ij}=\{\begin{array}{c} 1\;\;\;\;user\;i\;is\;associated\;with\;sBs_{j}\\ \;0\;\;\;\;\;\;\;\;\;\;\;\;\;otherwise \end{array}$$

where i=1, 2, …, K, and j=1, 2,…,n, *K*, and *n* are the total number of users and sBs in the cluster, respectively.

Each user is allowed to be associated with one sBs only and we assume that each user demands bitrate represented as a vector, $U_{BW}$, written as:

(2) $$U_{\mathrm{BW}}=\left[\begin{array}{ccc} U_{BW}^{1} & U_{BW}^{2}\ldots & U_{BW}^{\;K} \end{array}\right]$$

In LTE OFDMA, the smallest unit of resources that can be allocated per user is one resource block (RB) consisting of 1 time slot (7 symbols) and 12 subcarriers separated at 15 kHz spacing (180 kHz bandwidth) [17]. Thus, the total number of users associated with a serving sBs is upper bounded by the total effective capacity available in the sBs and the bit rate demanded by each user. The effective capacity perceived by user *K* from sBs *l* is expressed as:

(3) $$C_{k,l}=Blog_{2}\left(1+\gamma_{k,l}\right)$$

where B is the bandwidth available in sBs, and $\gamma_{k,l}$ is the signal to interference plus noise ratio (SINR) perceived by user K from sBs l, given as [20]:

(4) $$\gamma_{k,l}=\frac{p_{l}^{T}g_{k,l}}{\underset{j\neq l}{\sum_{j=1}^{n}}p_{j}^{T}g_{k,j}\rho_{j}+\sigma^{2}}$$

where $g_{k,j}$ is the channel gain between user K and sBs, l, $\rho_{j}$ are the neighboring cells load factor and can be interpreted as the probability that neighboring cells are transmitting at all sub-carrier bandwidths of serving sBs, $p_{l}^{T}$ and $p_{j}^{T}$ are the transmission power spectrum of resource block for serving and neighboring cells, and $\sigma^{2}$ is the noise power.

From Equations (2)–(4), the effective bitrate allocated for user *K* from the serving sBs *l* can be represented as:

(5) $$\rho_{kl}=\frac{U_{BW}^{k}}{Blog_{2}\left(\frac{p_{l}^{T}g_{k,l}}{\underset{j\neq l}{\sum_{j=1}^{n}}p_{j}^{T}g_{k,j}\rho_{j}+\sigma^{2}}+1\right)}$$

Thus, the load factor of active sBs *l* can be estimated by aggregating all its served users effective bit rates represented in Equation (5), mathematically [20]:

(6) $$\rho_{l}=\sum_{i=1}^{k}\frac{A_{m}^{il}U_{BW}^{i}}{Blog_{2}\left(\frac{p_{l}^{T}g_{i,l}}{\underset{j\neq l}{\sum_{j=1}^{n}}p_{j}^{T}g_{i,j}\rho_{j}+\sigma^{2}}+1\right)}$$

Each active base station load factor in Equation (6) is coupled with neighboring active sBs load factor [20]. Furthermore, the increase in neighboring active sBs load factors would result in an increase in the serving sBs load factor.

In mBs, the average achievable capacity for each user is given as, assuming zero-forcing precoding model, [5]:

(7) $${\hat{C}}_{K,o}=\left(1-\sum_{k=1}^{K}x_{k,0}\left(\frac{T^{\prime }}{T}\right)\right)\left(\frac{T_{u}}{T^{\prime }}\right)log\left(1+\frac{M_{0}-S_{0}+1}{S_{0}}\gamma_{k,0}\right)$$

where

(8) $$x_{k,0}=\{\begin{array}{c} 1\;\;if\;user\;k\;is\;served\;by\;mBs\\ 0\;\;\;\;\;\;\;\;\;otherwise \end{array},$$

$T,\;T^{\prime },\;$ and $T_{u}$ are the frame interval, interval of symbol, and useful symbol interval, respectively, and $T^{\prime }=T_{u}+T_{g}$, where $T_{g}$ is the guard interval,

$M_{0}$ is the number of antennas,
$S_{0}$ is the beamforming size, and
$\gamma_{k,0}$ is the SNR perceived by user k, the SNR is calculated through the channel state indicator.

Power consumption of each sBs consists of two parts, the first is the static power needed for cooling systems, power amplifier and baseband units, denoted as $P_{ol}$ for sBs *l*, and the last is the dynamic part and is related to the max transmission denoted as, $P_{l-max}^{Trans}$ [5], thus the total power consumed by sBs *l* is:

(9) $$P_{l}=P_{ol}+\rho_{l}\times P_{l-max}^{Trans}$$

While the total power consumed by all sBs in the cluster is the summation of Equation (8) over all active sBs:

(10) $$P_{T}=\sum_{l=1}^{n}P_{l}+\sum_{l=1}^{n}P_{ol}+\sum_{l=1}^{n}\rho_{l}P_{l-max}^{Trans}$$

In heterogenous networks, base stations have different static and dynamic power characteristics. In Equation (9), all the sBs are active and by introducing the average sBs load factor, $\bar{\rho }$, static power, $\bar{P_{o}}$, and the average maximum transmission power, $\bar{P_{max}^{Trans}}$, Equation (9) can be expressed as:

(11) $$P_{T}=n\bar{P_{o}}+n\bar{\rho }\bar{P_{max}^{Trans}}$$

where

(12) $$\bar{P_{o}}=\sum_{l=1}^{n}P_{ol}/n$$

(13) $$\bar{\rho }\bar{P_{max}^{Trans}}=\sum_{l=1}^{n}\rho_{l}P_{l-max}^{Trans}/n$$

In Equation (10), we assume that all sBs are active and operating at different load factors according to the user’s demand and number of served users. To maximize energy efficiency, sBs with low load factor are required to switch-off to reduce the total consumption power and the remaining active sBs are operating at full load or near full load factor. Thus, let us define the rate of active sBs as follows:

(14) $$s=\frac{\sum_{l=1}^{n}y_{l}}{n}$$

where

(15) $$y_{l}=\{\begin{array}{c} 1\;is\;sBs_{l}\;is\;active\\ 0\;\;\;\;\;\;\;\;otherwise \end{array}$$

Let us further assume that the inactive sBs is consuming average power denoted as, $\bar{P_{sw}}$, which is less than $P_{o}$ consumed by the active sBs, that is:

(16) $$\bar{P_{sw}}<\bar{P_{o}}$$

During the operation of the network, the total power consumed from active sBs would be $ns\bar{P_{o}}$, and the inactive sBs would consume $n\left(1-s\right)\bar{P_{sw}}$. Further, the inactive sBs would not have load factor, $\rho_{l}=0,\;if\;y_{l}=0$. Thus, if s percentage of sBs are inactive, then Equation (10) would be written as:

(17) $$P_{T}=ns\bar{P_{o}}+n\left(1-s\right)\bar{P_{sw}}+ns\bar{\rho }\bar{P_{max}^{Trans}}$$

To this end, EE in massive MIMO systems can be defined as the total capacity perceived by users served by sBs’s and mBs, to the total power consumed in the sBs. Thus, from Equations (3), (7) and (13), EE is represented as:

(18) $$E=\frac{{\hat{C}}_{K,o}\sum_{i=1}^{K}A_{m}^{i,0}+\sum_{l=1}^{n}y_{l}C_{k,l}\sum_{j=0}^{K}A_{m}^{i,l}}{ns\bar{P_{o}}+n\left(1-s\right)\bar{P_{sw}}+ns\bar{\rho }\bar{P_{max}^{Trans}}}$$

(19) $$EE=\frac{{\hat{C}}_{K,o}\sum_{i=1}^{K}A_{m}^{i,0}+ns{\hat{C}}_{K,l}\sum_{j=0}^{K}A_{m}^{i,l}}{ns\bar{P_{o}}+n\left(1-s\right)\bar{P_{sw}}+ns\bar{\rho }\bar{P_{max}^{Trans}}}$$

(20) $$\bar{\rho }=as$$

where a is constant positive.

Then, $\frac{\bar{\rho }}{s}=a$

(21) $$\frac{\left(d\bar{\rho }/dt\right)s-\left(ds/dt\right)\bar{\rho }}{s^{2}}=0$$

(22) $$\left(d\bar{\rho }/dt\right)s-\left(ds/dt\right)\bar{\rho }=0$$

(23) $$\bar{\rho }=\frac{\left(d\bar{\rho }/dt\right)}{\left(ds/dt\right)}s=\frac{d\bar{\rho }}{ds}s$$

(24) $$EE=\frac{{\hat{C}}_{K,o}\sum_{i=1}^{K}A_{m}^{i,0}+ns{\hat{C}}_{K,l}\sum_{j=0}^{K}A_{m}^{i,l}}{ns\bar{P_{o}}+n\left(1-s\right)\bar{P_{sw}}+ns^{2}\frac{{\bar{\rho }}^{,}}{s^{\prime }}\bar{P_{max}^{Trans}}}$$

(25) $$EE=\frac{{\hat{C}}_{K,o}s^{\prime }\sum_{i=1}^{K}A_{m}^{i,0}+nss^{\prime }{\hat{C}}_{K,l}\sum_{j=0}^{K}A_{m}^{i,l}}{ns^{\prime }\left(s\bar{P_{o}}+\left(1-s\right)\bar{P_{sw}}\right)+ns^{2}{\bar{\rho }}^{\prime }\bar{P_{max}^{Trans}}}$$

Further, we assume that users are either served by sBs or mBs, thus, the total number of served users is:

(26) $$\sum_{i=1}^{K}A_{m}^{i,0}+\sum_{j=0}^{K}A_{m}^{i,l}=K$$

(27) $$K_{\mathrm{mBs}}=K-K_{\mathrm{sBs}}$$

where

(28) $$K_{mBs}=\sum_{i=1}^{K}A_{m}^{i,0},\;\mathrm{and}\;\;K_{sBs}=\sum_{j=0}^{K}A_{m}^{i,l}$$

The equations presented aim to construct a mathematical representation and enhance the energy efficiency of the massive MIMO network. This is achieved by taking into account several elements such as user association, resource allocation, and power consumption. This type of study holds significant value in the realm of creating and overseeing efficient wireless communication networks. The key parameters and variables integral to the proposed optimization framework are presented in Table 1.

Our proposed multi-base station massive MIMO power model delves into the pivotal elements of our energy efficiency framework for managing massive MIMO base stations. Central to this approach is the Dynamic Adjustment of base station operations, which aligns with real-time network demands, thus minimizing energy consumption during periods of low traffic. The heart of our framework is an advanced Optimization Algorithm designed specifically for Power Minimization. This algorithm optimally selects active base stations and adjusts load factors to achieve an equilibrium between energy use and network performance. Additionally, through comprehensive Energy Consumption Comparisons, our simulations reveal that our method significantly outperforms traditional base station management techniques in terms of energy efficiency. This is particularly evident in scenarios with varying base station activity levels and load distributions. These features collectively highlight the innovative and sustainable approach of our framework in enhancing the efficiency of massive MIMO networks.

## 3. Results and Dissection

In this study, we evaluated the energy efficiency (EE) of a massive MIMO network using the simulation approach. Our focus was on understanding how the proportion of active base stations (s) and the load factor (ρ) affect the network’s EE. The experimental setup and simulation methodology used to validate the proposed optimization framework in your study involve a MATLAB simulation environment. The key parameters include 100 base stations and 1000 users. The simulation varies the proportion of active base stations (ranging from 50% to 90%) and employs fixed load factor and power consumption values for active and switched-off small base stations. The simulation randomly generates user capacities from both macro and small base stations and calculates EE for each scenario. The performance metrics used are EE and the number of active small base stations, plotted to visualize the relationship between base station activity and energy efficiency. The simulation parameters were carefully chosen to reflect realistic operating conditions for a massive MIMO network, as outlined in Table 2.

**Figure 2:**
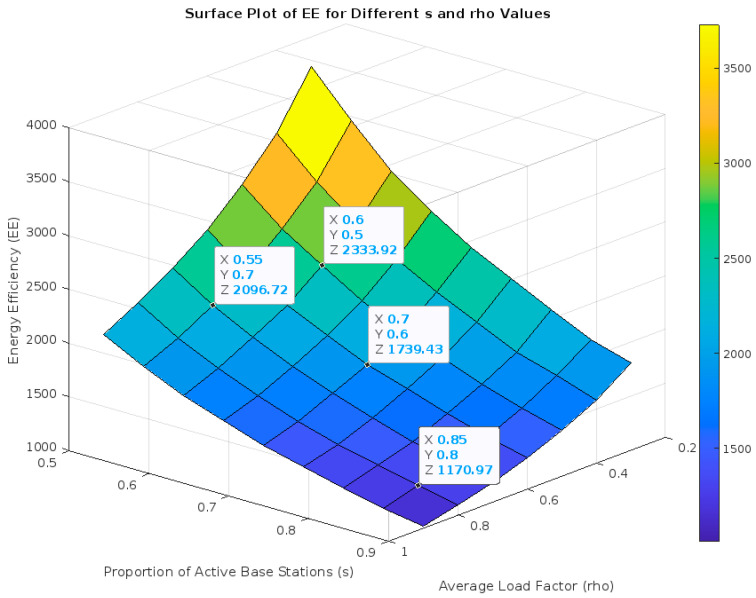
Energy Efficiency (EE) for Different $s^{\prime }$ and $\bar{\rho }$ Values.

The simulation results, presented in Figure 2, reveal a complex relationship between $s^{\prime }$, $\bar{\rho }$ and EE. As expected, we found that EE is affected by the proportion of active base stations and the load factor. Specifically, EE tended to decrease when the proportion of active base stations increased, underlining the impact of power consumption by active base stations on the network’s overall energy efficiency. Conversely, higher load factors tended to increase the energy efficiency, up to a point where the system became saturated, and the efficiency gains leveled off.

**Figure 3:**
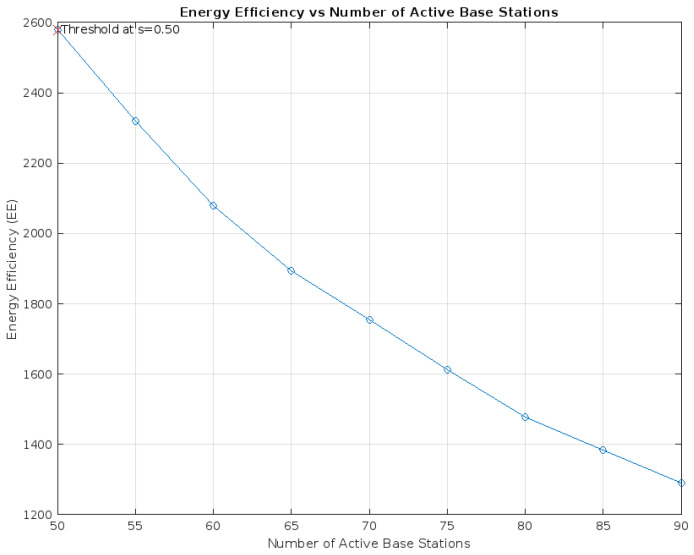
Energy Efficiency (EE) vs. Number of Active Base Stations.

Furthermore, the plot of EE against the number of active Base Stations (Figure 3) shows Energy Efficiency (EE) decreasing as the number of active base stations increases from 50 to 90. This aligns with the paper’s discussion on the power dynamics in massive MIMO systems, where increasing the number of active base stations leads to higher overall power consumption, thus reducing EE. The plot of Energy Efficiency (EE) against the number of active base stations, as depicted in Figure 3, reveals a decline in EE with the increase in active stations, supporting the paper’s discussion on power dynamics in massive MIMO systems. Interestingly, EE improvements appear to plateau beyond a certain threshold, highlighting a pivotal balance between energy savings and network capacity. This observation underscores the necessity of achieving an optimal mix of active base stations and load factors for maximized energy efficiency. However, it is crucial to integrate considerations of guaranteed service availability into this equation, ensuring that efficiency gains do not come at the expense of network reliability and user experience. This balance is essential for network operators aiming to optimize energy use without compromising service quality.

**Figure 4:**
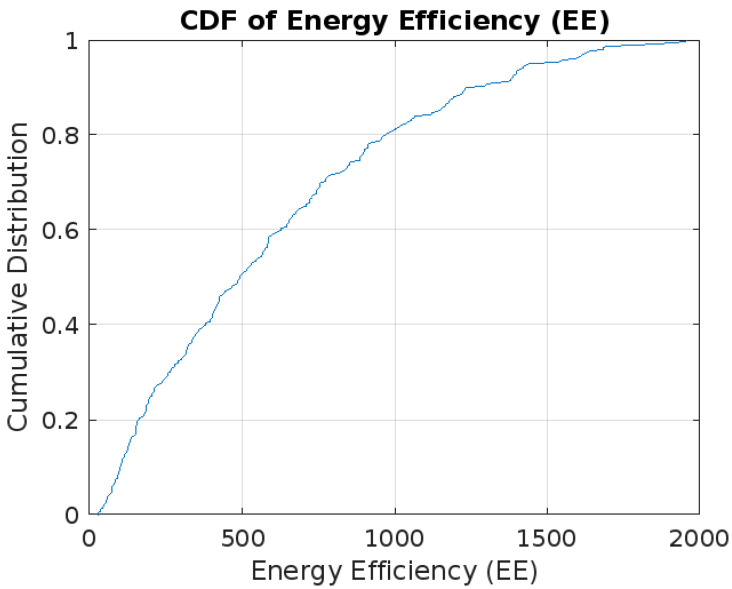
CDF of Energy Efficiency (EE).

Figure 4 shows the cumulative distribution function (CDF) of Energy Efficiency (EE) values exhibits a steady ascent, reflecting a uniform spread across the observed range without abrupt fluctuations or outliers. The CDF of Energy Efficiency (EE) in a massive MIMO network reveals crucial insights into the distribution and variability of EE values. The CDF plot helps identify where the majority of EE values are concentrated; a steeper curve suggests most configurations cluster around lower EE values. The curve’s shape also indicates the degree of variability in EE across configurations, with a steeper initial slope pointing to less variability. Understanding these patterns is essential for gauging common EE levels and the likelihood of achieving various efficiency outcomes in network configurations.

The comparative analysis of our model with two studies [6,21], which used reinforcement Learning in massive MIMO networks underscores different optimization strategies. Our model focuses on a mathematical approach to balance active base stations and load factors for enhanced energy efficiency. Meanwhile, the other studies apply Reinforcement Learning for antenna switching, with one achieving significant energy efficiency gains through User Equipment location data and the other employing a Radio Environment Map to optimize antenna activity. This contrast highlights the diversity of techniques in the field of massive MIMO network optimization. While our study presents a comprehensive approach, it is important to acknowledge potential limitations. The practical implementation of our proposed model might face challenges in real-world, dynamic network environments. Scalability and adaptability to rapidly changing network conditions, such as user mobility and fluctuating traffic patterns, are also significant concerns. Future research could focus on integrating machine learning techniques for more dynamic optimization and conducting real-world case studies to validate and refine our model across various network scenarios. Addressing these limitations will enhance the practical applicability and robustness of our optimization framework.

## 4. Conclusions

This study has focused on the crucial matter of energy efficiency in massive MIMO networks, which play a crucial role in satisfying the increasing demands of upcoming wireless services. The utilization of massive MIMO technology, although holding significant potential for achieving very high capacity, is intrinsically characterized by a substantial energy consumption, hence necessitating the implementation of energy-efficient operational practices. In order to address this particular obstacle, we have phrased the problem of energy efficiency in massive MIMO networks as a restricted variational problem. The method we have proposed exhibits both uniqueness and boundedness in its solutions, while also offering a thorough framework for optimizing total energy. Furthermore, a strategic strategy for switching base stations on and off was developed in order to minimize the signaling required for handovers and to guarantee equitable distribution of user capacity. The findings of our research demonstrate the efficacy of variational optimization in attaining significant reductions in energy consumption while maintaining user capacity demands. In addition, the base station selection criteria that we have provided have resulted in a reduction in handover overhead signaling, hence significantly boosting the overall efficiency of the system. Our research is a valuable contribution to the expanding field of knowledge concerning energy-efficient operations in massive MIMO networks. It presents a practical method for optimizing base station switching without compromising the quality of wireless services. The energy-efficient solutions outlined in this study demonstrate considerable potential for fostering a sustainable and resource-conscious future in wireless communication networks, as the demand for wireless connectivity continues to experience substantial growth.

## Figures and Tables

**Table 1:** Key parameters and variables used in the proposed optimization framework.

Parameter	Description
*mBs*	Macro Base Station, with multiple antennas
*sBs*	Small Base Stations, typically micro base stations
$A_{m}^{ij}$	Association matrix, defining user-base station associations
$U_{\mathrm{BW}}$	User Bitrate Vector
$C_{k,l}$	Effective capacity perceived by user k from sBs l
$\gamma_{k,l}$	Signal to Interference plus Noise Ratio (SINR)
$\rho_{kl}$	Effective bitrate allocated for user k from sBs l
$\bar{\rho }$	Load factor of active sBs
${\bar{\rho }}_{o}$	Static power needed for systems like cooling in sBs
$\bar{P_{max}^{Trans}}$	Maximum transmission power for sBs
EE	Energy Efficiency

**Table 2:** Simulation parameters.

Parameter	Description	Value(s)
n	Number of base stations	100
K	Number of users	1000
$s^{\prime }$	Proportions of active base stations	0.5, 0.6, 0.7, 0.8, 0.9
$\bar{\rho }$	Load factor	0.6
$\bar{P_{o}}$	Static power consumption of active sBS	100 Watts
$P_{sw}$	Power consumption of switched off sBS	50 Watts
$P_{max}^{Trans}$	Max transmission power of sBS	200 Watts
${\hat{C}}_{k,0}$	Capacities for users from mBS	Random up to 100 Mbps
$C_{k,l}$	Capacities for users from each sBS	Random up to 10 Mbps

## Data Availability

The original contributions presented in the study are included in the article.
